# Proteomics: a biotechnology tool for crop improvement

**DOI:** 10.3389/fpls.2013.00035

**Published:** 2013-02-28

**Authors:** Moustafa Eldakak, Sanaa I. M. Milad, Ali I. Nawar, Jai S. Rohila

**Affiliations:** ^1^Department of Biology and Microbiology, South Dakota State UniversityBrookings, SD, USA; ^2^Department of Genetics, Faculty of Agriculture, El Shatby, Alexandria UniversityAlexandria, Egypt; ^3^Biotechnology Lab, Department of Crop Science, Faculty of Agriculture, El Shatby, Alexandria UniversityAlexandria, Egypt

**Keywords:** biotechnology, crop improvement, proteomics, sustainable agriculture

## Abstract

A sharp decline in the availability of arable land and sufficient supply of irrigation water along with a continuous steep increase in food demands have exerted a pressure on farmers to produce more with fewer resources. A viable solution to release this pressure is to speed up the plant breeding process by employing biotechnology in breeding programs. The majority of biotechnological applications rely on information generated from various -omic technologies. The latest outstanding improvements in proteomic platforms and many other but related advances in plant biotechnology techniques offer various new ways to encourage the usage of these technologies by plant scientists for crop improvement programs. A combinatorial approach of accelerated gene discovery through genomics, proteomics, and other associated -omic branches of biotechnology, as an applied approach, is proving to be an effective way to speed up the crop improvement programs worldwide. In the near future, swift improvements in -omic databases are becoming critical and demand immediate attention for the effective utilization of these techniques to produce next-generation crops for the progressive farmers. Here, we have reviewed the recent advances in proteomics, as tools of biotechnology, which are offering great promise and leading the path toward crop improvement for sustainable agriculture.

## INTRODUCTION

**FIGURE 1:**
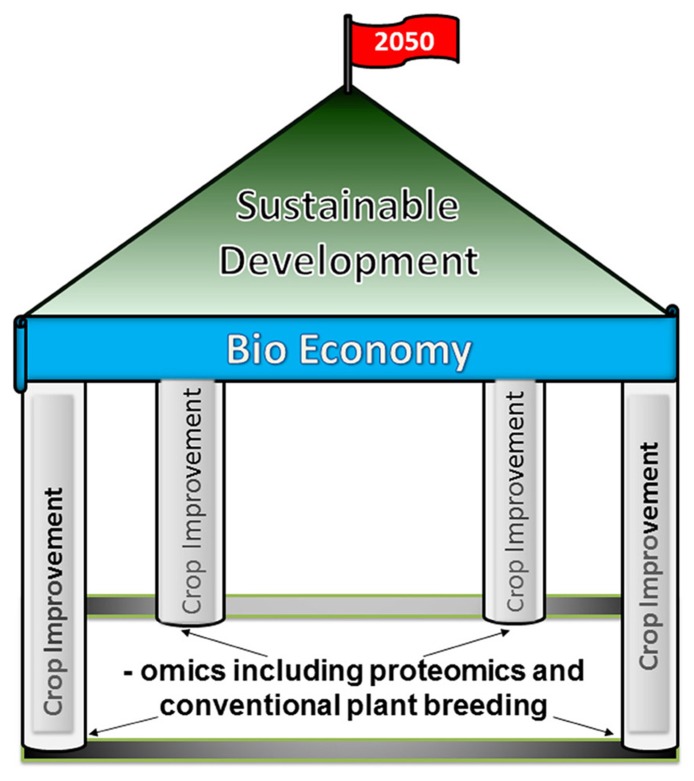
**An illustration showing that the -omics and the conventional plant breeding techniques are the pillars of bio-economy, and a strong bio-economy is the foundation of sustainable development of a society**. By the incorporation of these technologies for crop improvements, the bio-economy is uplifted and thus, we should be able to reach our strategic goals set for the agricultural productions by the year 2050.

According to an estimate, there are approximately 925 million people on the globe who live in a state of hunger (Karimizadeh et al., 2011). Moreover, an additional two billion people are expected to be added by the year 2050 (UN, 2012). In an effort to eradicate that ugly spot of hunger from the beautiful face of the humanity, we need to significantly increase the production and supply of food by integrating different elements and strengthening the plant breeding tools (Beddington et al., 2012) for crop improvements. A major hurdle for crop improvement programs faced by the plant breeders is a limited gene pool of domesticated crop species. The identification of potential useful genes across the animal and plant kingdom that could play key roles toward the improvement of important crop traits, generally derived from research in molecular biology including genomics and proteomics, is a crucial step. Such newly discovered genes, when placed into a desired crop species and then utilized for breeding programs, could be a boon to human society (**Figure 1** and **Table 1**).

**Table 1:** A short overview of recent gel-based and gel-free proteomics methods as biotechnological tools that could provide knowledge for crop improvement programs.

Major crops	Technique used	Trait studied	Plant part	Reference
Wheat	2-DE	Desiccation	Embryo	Irar et al. (2010)
	iTRAQ and 2D-DIGE	Drought	Leaves	Ford et al. (2011)
	2D-DIGE	Salinity	Leaves	Gao et al. (2011)
	2-DE	Senescence and oxidative stress	Stem	Bazargani et al. (2011)
	2-DE	Flooding stress	Root	Kong et al. (2010)
	2-DE	Metabolism post anthesis	Endosperm amyloplast	Dupont (2008)
	2-DE	*Fusarium* head blight	Kernels	Foroud et al. (2008)
	2-DE	Heat	Kernels	Laino et al. (2010)
Maize	2-DE	Unintended effects of GM	GM vs. non-GM leaves	Barros et al. (2010)
	nanoLC-LTQ-Orbitrap	C_4_ leaf development	Leaves	Majeran et al. (2010)
	2-DE	Desiccation	Embryo	Huang et al. (2012)
	Shotgun proteomics	Photosynthesis	Chloroplast thylakoid membrane	Liu et al. (2011)
	Shotgun proteomics	Desiccation	Embryo	Amara et al. (2012)
	2-DE	Drought	Xylem sap in root and stem	Alvarez et al. (2008)
	iTRAQ	Ear rot infection	Ears	Mohammadi et al. (2011)
	LC-MS	Greening of etiolated leaves	Leaves	Shen et al. (2009)
Soybean	2-DE	Tolerance to *Phytophthora*	Hypocotyls	Zhang et al. (2011b)
	2-DE and blue native PAGE	Flooding stress	Roots and hypocotyl	Komatsu et al. (2011)
	2-DE	Oxidative stress	Leaves	Galant et al. (2012)
	2-DE	Heat stress	Leaves	Wang et al. (2012)
	2-DE	Flooding stress	Roots, Hypocotyl, and leaves	Khatoon et al. (2012)
	2-DE	Osmotic stress	Roots	Toorchi et al. (2009)
	iTRAQ	Enhancing water and nutrient uptake after inoculation with Bradyrhizobium	Root	Nguyen et al. (2012)
Rice	2-DE	Response to selenium	Leaves	Gong et al. (2012)
	2-DE	Embryogenesis	Embryo	Zi et al. (2012)
	Shotgun proteomics	Grains development	Grains	Lee and Koh (2011)
	2-DE	Heat stress	Spikelet	Jagadish et al. (2010)
	2-DE	Drought stress	Rice peduncles	Muthurajan et al. (2011)
	iTRAQ	Cold stress	Leaves	Neilson et al. (2011)
	2-DE	Bacterial blight defense signaling	Leaves	Mahmood et al. (2009)

Various completed and several ongoing plant sequencing projects, e.g., *Arabidopsis thaliana *(Arabidopsis Genome Initiative, 2000), rice (Goff et al., 2002; Yu et al., 2002), and soybean (Schmutz et al., 2010) are reaching the phase where they can provide the blueprints to modern breeders to access a great number of genes, but the benefits derived from these blueprints can only be harvested when the spatial and temporal expressions, functions and interactions of the gene products become well-characterized (Salekdeh and Komatsu, 2007). In simple words, the genetic (DNA) information of a plant is translated via the intermediary step of transcription (mRNA) into a protein. One of the several cutting edge approaches for understanding global genes expression and their functional mechanisms is to study the proteins translated from those genes and that scientific branch is known as proteomics. The word “proteome” is derived from PROTEins expressed by a genOME. Analogous to genomics, the term “proteomics” describes the study and characterization of the complete set of proteins present at a given time in the cell (Wilkins et al., 1995). Although genomic studies are helpful to scientists in knowing what is possible theoretically, proteomic studies reveal the functional players for mediating specific cellular processes. The proteome, unlike the genome, which is static in nature, has dynamic capabilities. The study of proteins introduces post-translational modifications (PTMs) and provides the knowledge that is important for understanding the biological functions (Nat et al., 2007). The PTMs that play important roles during the growth and development of a plant and/or in response to various stress conditions cannot be understood from the genome sequence projects and/or transcript abundance alone. Proteomics knowledge provides functional genomics with a completeness toward understanding the process (Gygi et al., 2000; Dubey and Grover, 2001; Park, 2004; Thurston et al., 2005). Furthermore, the development of various advanced tools for bioinformatics and computational science are connecting proteomics to other “-omics,” and the physiological data are further opening up new methods for crop improvement studies via the signaling, regulatory, and metabolic networks underlying plant phenotypes (Kitano, 2002; Langridge and Fleury, 2011).

Similar to proteomics, the biotechnology field has seen advancements in the past several decades (Varshney et al., 2011). Comparatively, it is now a fully mature science and is proud to be on the list of most quickly adopted crop technologies in world. Biotechnology provides the capabilities to breeders to achieve certain goals that would otherwise be impossible through conventional plant breeding approaches. Globally, today genetically modified crops are grown in fields at a commercial scale. Thus, the biotech crop area has increased from 1.7 million ha in 1996 to 160 million ha in 2011 (Khush, 2012). This trend was well-expected by Dixon (2005) when he stated that “Genomics (originally DNA- and transcript-based, but recently extended to integrate the proteome and metabolome) would play a major role in driving plant biotechnology.” This review corroborates his long vision and focuses on the use of proteomics for genetic improvements in food and biofuel crops including food quality, safety, and nutritional values, tolerance to abiotic and biotic stresses, manufacturing plant-based vaccines and proteomics-based fungicides. Apart from these, proteomics is being used for several other crop improvement programs such as, pre- and post-harvest losses, and crop quality characteristics but that is not a part of this review because of space constraints.

## PROTEOMIC TECHNIQUES OFFER NEW TOOLS FOR PLANT BIOTECHNOLOGY

The knowledge of key proteins that play crucial roles in the proper growth and development of a plant are critical to propel the biotechnological improvement of crop plants. These proteins maintain cellular homeostasis under a given environment by controlling physiological and biochemical pathways. A search of the published research literature revealed that genomics and proteomics are the two major wheels that keep the discovery of novel genes rolling, which can eventually be placed into the pipeline for crop improvement programs. Two-dimensional electrophoresis (2-DE) and mass spectroscopy (MS), two of the most widely used proteomics methods, are used to catalog and identify proteins in different proteome states or environments. Advances in 2-DE have been extremely helpful in bringing proteomics close to biotechnological programs; however, due to some drawbacks and disadvantages associated with gel-based proteomics, e.g., labor intensiveness, insensitiveness to low-copy number proteins, low reproducibility and the inability to characterize complete proteomes, many gel-free proteomic techniques have also become a valuable tool for scientists (Baggerman et al., 2005; Lambert et al., 2005; Scherp et al., 2011; Jayaraman et al., 2012).

## POTENTIAL OF PROTEOMICS AS A BIOTECHNOLOGY TOOL IN CROP IMPROVEMENT PROGRAMS

### MOLECULAR MARKERS ARE TO ASSIST PLANT BREEDERS

Proteomics offers novel gene (DNA) identifications to plant biologists and breeders. Marker-assisted selection (MAS), which is the employment of DNA markers in a plant breeding program, has extensively been used to select desired genes/quantitative trait loci (QTLs) in the development of a comparatively superior breeding line (Collard and Mackill, 2008). Damerval et al. (1994) used an approach that brought proteomic and MAS components together; they identified protein quantity loci (PQL) that explained some of the spot intensity variation. Of the 72 proteins analyzed, 70 PQLs were identified for 42 proteins, 20 of which had more than one PQL. This type of approach is especially useful in breeding programs because, through intensive breeding selection, lines could be available with differing phenotypic degrees that help in drawing correlations between responsive genes and observed stress tolerance phenotypes. This correlation can further be verified by analyzing advanced mapping populations such as recombinant inbred lines (RILs), near isogenic lines (NILs), and double haploid lines (Salekdeh and Komatsu, 2007). Furthermore, the co-segregation of a protein and the QTL (or the trait) can be studied in the two parental lines from which the mapping populations were developed. Finally, the plant breeders should be able to integrate the selected genes in marker-assisted breeding programs to improve the trait under study (Salekdeh and Komatsu, 2007). The major limitation of this technique is that it works only with-in the same species because the parents need to be cross-compatible to transfer the superior genes/alleles through this molecular breeding approach. Under such limitations, embryo rescue or genetic engineering, which has no boundaries for gene transfer, could be very useful (Varshney et al., 2011).

### CHARTING THE PROTEOME AND ITS INTERACTION MAP IS IMPORTANT FOR CREATING A KNOWLEDGE BASE

As a general statement, almost all cultivated lands fall under sub-optimal conditions for commercial agriculture (Komatsu, 2008). Due to these sub-optimal conditions, up to 70% of the crop yields could be lost (Boyer, 1982). To increase crop productivity, genes and proteins that are responsible for stress tolerance and disease resistance have to be identified continuously. In this direction, a snapshot of the cellular proteome map at a given time and under given conditions facilitates the identification of changes in protein expression (Hashiguchi et al., 2010). Advancements in MS-based proteomics platforms have been considered to be “New Genomics” because MS has become an indispensable tool for the investigation of the PTMs to proteins, and protein interactions. These data provide an unprecedented insight into how cells make decisions and are thus a cornerstone of systems biology (Cox and Mann, 2007). None-the-less, the knowledge about the interacting protein partners, essential for the success of the function of a particular protein, might be a good target for gene pyramiding in species that lack the interacting protein(s). In the recent past, several successful projects have been completed to create proteome maps of various crops using 2-DE and/or other proteomic approaches. For example, in wheat a reference map has been created for leaves (Donnelly et al., 2005), roots (Song et al., 2007), endosperm (Vensel et al., 2005), and amyloplasts (Balmer et al., 2006). This knowledge is helping us to understand the biological processes that occur in these plant organs. Rice, a staple food for more than half of the world’s population (Narciso and Hossain, 2002), witnessed a boom in proteomics studies soon after its genome was sequenced (Agrawal and Rakwal, 2006; Rohila et al., 2009; Roy et al., 2011). A significant knowledge database has already been made in rice toward identifying and cataloging the proteins from various tissues and organelles. This knowledge is in the pipeline and waiting to be used by biotechnologists and molecular plant breeders.

### PROTEOMICS HELP THE INVESTIGATIONS OF ABIOTIC AND BIOTIC STRESS TOLERANCE MECHANISMS

As with any living organism, crop plants also have to cope with various biotic and abiotic stress conditions. Contrary to greenhouse nurseries, plants in the field experiences a combination of various biotic and abiotic stresses either concurrently or at different developmental stages throughout the growing season (Tester and Bacic, 2005; Mittler, 2006).

A recent estimate suggested that the increased temperatures of the past two decades have caused a loss of approximately $5 billion by impacting the yields of major food crops such as wheat, rice, maize, and soybeans (Peng et al., 2004). Temperatures reaching 35°C in the field cause rice and maize to show sterility. Such high heat conditions in the field also lead to flowering and fruiting failure in other crops. Molecular plant physiologists know very well that heat stress increases membrane damage and impairs metabolic functions (Taiz and Zeiger, 2010). A plant breeder needs to activate the proper protection systems in a crop plant to enable the survival of the plant’s cells under such heat stress conditions. Heat stress tolerance is a complex mechanism and is controlled by multiple genes and proteins involving a number of physiological and biochemical changes in the cell, e.g., adjustments in the membrane structure and function, tissue water content, protein composition, lipids, and primary and secondary metabolites (Huang and Xu, 2008). Global proteomic profiling projects are useful techniques for increasing the knowledge base of plant breeders. For example, a study comparing various wheat cultivars with different heat tolerance capabilities revealed low molecular weight (16–17 kDa) heat shock protein (HSPs) and other metabolic proteins crucial for the heat tolerance phenotype (Majoul et al., 2004). Proteins from the HSP family and the transcription factors upstream of these HSPs have been found to have crucial roles in providing thermotolerance to the crop. Disarming the function of HSP100 by introducing an antisense construct in tomato plants resulted in their poor survival under heat stress conditions (Yang et al., 2006). However, in another study, transgenic lines overexpressing a different HSP protein (HSP70) showed superior thermotolerance in soybean plants (Zhu et al., 2006). Furthermore, protein–protein interaction studies have proved that HSP90 interacts with calmodulin-binding protein (CBP) (Virdi et al., 2009). Thus, the studies by Zhang et al. (2009) showed that the knockdown of calmodulin resulted in reduced thermotolerance. Proteins other than HSPs, e.g., CBP in the above study, have been identified in other proteomic studies as differentially expressed proteins during heat stress conditions. Süle et al. (2004) proposed S-adenosylmethionine synthetase as a molecular marker for screening heat-tolerant germplasms. Even with this information, knowledge on the systemic response of plants during heat stress remains limited because plant perception and response to a single stress is different than to a combination of multiple stresses.

There is another major constraint to world agriculture in the form of limited water availability for crop irrigation. Recent climate variability from year to year predicts a worsening situation in the future. World climatologists predict that global warming will result in more frequent and severe droughts in the coming years. Drought stress causes a decrease in carbon usage by the photosynthetic machinery that result in net yield losses on the farm. Physiological experiments have shown that drought conditions inhibit plant photosynthesis within a short time of a limited water supply resulting in a drop in the CO_2_ assimilation rates (Ribas-Carbo et al., 2005). To minimize water loss, plants need to close their stomata under water deficient conditions. The guard cells help the plant in the process of controlling the opening and closing of the stomata. The closure/opening of the stomata is controlled by the plant hormone, abscisic acid (ABA). In a plant cell, ABA flux concentrations are controlled in response to the availability of water to the plant. ABA has been found to play an indispensable role in the plant response to drought conditions by inducing many transcription factors. In this direction, the guard cell proteome profiling by Zhao et al. (2008) revealed 336 proteins responsive to water stress conditions, with a further 52 proteins considered to be signaling proteins. Abiotic stresses in general cause a water deficit condition in cells that results in a myriad of complex cellular and physiological responses at the plant cellular and organismal levels. In general, the net photosynthesis rate is reduced either because of stomatal closure or via metabolic impairment (Reddy et al., 2004). The changes in mitochondrial respiration and the photosynthetic electron transport chain lead to the generation of highly toxic reactive oxygen species (ROS), such as superoxides and peroxides, and cause chemical damage to the DNA and proteins. This damage has serious effects on cellular metabolism (Mittler, 2002). During evolution, plants have developed several strategies to address ROS, e.g., avoidance by anatomical adaptation, photosynthesis suppression and photosystem and antenna protein complex modulations. Several metabolites, such as ascorbate and glutathione, and enzymes, such as peroxidases and superoxide dismutases, help to scavenge the ROS (Mittler, 2006). Another plant strategy to address drought conditions is to maintain the turgor pressure of plant cells by the overproduction of osmolytes, such as proline, glycine betaine, and trehalose. These metabolites provide secondary protective effects to proteins against misfolding (Hare et al., 1998). Moreover, dehydration responsive proteins, such as dehydrins and HSPs, are over produced to protect the intracellular metabolic machinery (Wang et al., 2003). In short, with such a wealth of knowledge, drought-tolerant plants can be generated by the modification of these mechanisms, e.g., ABA signaling can be adjusted for the better survival of a crop plant under such stress conditions. The level of sphingosine-1-phosphate, a messenger molecule, is controlled by ABA through the sphingosine kinase protein. In another study using a sphingosine-1-phosphate lyase mutant, the accumulation of sphingosine-1-phosphate decreased the fresh weight loss of plants under drought stress conditions by controlling water loss from the stomata (Nishikawa et al., 2008). Hajheidari et al. (2005) report the predominance of proteins that are related to ROS management and protein stability after investigating the proteomic profiling of field-grown plants under drought stress conditions.

Proteomic approaches are useful in the study of the molecular mechanism involved in the interaction between a plant and its pathogens (Zhou et al., 2006). This group inoculated the wheat spikelet with the fungal spores of *Fusarium graminearum* and subjected the total proteins from the infected spikelet to 2-DE for proteome profiling under normal and infected conditions. They discovered that 41 proteins were differentially regulated due to *Fusarium *infection. The gene ontology (GO) annotation revealed that the up-regulated proteins were from the antioxidant and JA signaling pathways, pathogenesis-related response, amino acid synthesis and nitrogen metabolism, whereas the down-regulated proteins were from the photosynthesis pathway. A DNA-damage inducible protein was found to be up-regulated and glycosylated (a type of PTM) in a *Fusarium*-infected spikelet. Furthermore, utilizing the TargetP software, several identified plant proteins were predicted to localize to the chloroplast. This knowledge further strengthened the previous finding that the chloroplast is the organelle most affected by *Fusarium* infections. Several fungal proteins were also identified and found to possess antioxidant and carbon-acquiring functions from the plant through the glycolysis reaction during a compatible interaction between *Fusarium* and the plant. Studying the proteome response of the resistant wheat cultivar Wangshuibai, Wang et al. (2005) found that expression of the carbon metabolism and photosynthesis genes decreased significantly after 6, 12, and 24 h of spike inoculation with the fungus *Fusarium*. In a separate study, the global proteomic analysis of germinating maize embryos after infection with *Fusarium verticillioides* highlighted the contribution of protein synthesis, protein folding, and stabilization, and oxidative stress tolerance proteins (Campo et al., 2004). Chivasa et al. (2005) studied a maize cell suspension culture with pathogen elicitors and showed that the responses to the pathogen attacks were localized to the extracellular matrix. The elicitor treatment of the cell cultures induced a rapid change in the phosphorylation status of extracellular peroxidases, the disappearance of the putative extracellular b-*N*-acetylglucosaminidase, and the accumulation of the putative secreted xylanase inhibitor protein. The accumulation of glyceraldehyde-3-phosphate dehydrogenase and a fragment of a putative HSP were observed at the start of the defense response time. Konishi et al. (2001) identified protein expression changes in rice leaves infected with the blast fungus *Magnaporthe grisea*. They found a correlation between quantitative expression changes in blast responsive proteins and the amount of applied nitrogen fertilizer. The large and small RuBisCO subunits were among the proteins that were increased by the nitrogen applications, whereas the small RuBisCO subunit was reduced after a nitrogen application and *Magnaporthe* infection. After the *Magnaporthe* infection, PR1 was among the proteins that were induced by the nitrogen application. Based on the results of this study, these proteins were proposed to potentially be involved in the incompatible interactions between the plants and the fungus and thus might be good candidates for approaching through plant biotechnology.

Proteins from the rice plasma membrane were studied by Chen et al. (2007), who analyzed the early defense responses involved in Xa21-mediated disease resistance. *Xa21* is a receptor kinase in rice, and is predicted to detect the pathogen (*Xanthomonas*) signal on the cell surface. In this investigation, 20 proteins were found differentially expressed by *Xanthomonas* infection after 12 and 24 h of inoculation. Eight of these proteins were plasma membrane-associated proteins and had potential functions in rice defense, whereas two proteins were not associated with the plasma membrane. By comparing two partially resistant lines with a susceptible control tomato line over time (72 and 144 h post-inoculation), plant proteins were found to be regulated in response to *Clavibacter michiganensis ssp. *Michiganensis infection. Using a 2-DE approach, 26 differentially regulated plant proteins were discovered, with 12 being stress response proteins and related to defense protein families (PR3 and PR9; Coaker et al., 2004). The resistant tomato line showed the up-regulation of *PR3, SOD, thioredoxin*, and *S-adenosylhomocysteine hydrolase* genes. In *Medicago truncatula*, a global proteomic analysis was used to characterize the plant response to the pathogenic bacterium *Pseudomonas aeruginosa *(Mathesius et al., 2003). The study established that 154 proteins were accumulated upon exposure to *P. aeruginosa*, with 21 of those proteins reported to be related to the defense and stress response mechanisms. Afroz et al. (2009) reported the differential expression of proteins in bacterial wilt-sensitive and wilt-resistant tomato cultivars using 2-DE and Edman sequencing. Molecular chaperones and proteins related to defense storage were highly expressed in the resistant cultivars compared with the susceptible cultivars.

All of the studies described above, and many that are not included here because of space limitations, are decent examples that prove that proteomics is highly capable of discovering novel genes/proteins that could be potential candidates for further studies via biotechnological approaches. We hope that, with time, the data sets for crop proteomics will strengthen further and that we will be able to see examples in which such proteomic-based knowledge is used directly for the improvement of the stress tolerance of a crop plant (Agrawal et al., 2012).

### MANUFACTURING PLANT-BASED VACCINES IS A POSSIBILITY IN NEAR FUTURE

An antigen of interest, when overexpressed in plant tissues by a biotechnological approach, is considered to be a plant-based vaccine (Chargelegue et al., 2001). In situations dealing with a poorly characterized pathogen, a genomic or proteomic approach is specifically useful to identify the candidate antigens that possess favorable characteristics (Scarselli et al., 2005; Streatfield, 2005). A major advantage of plant-based vaccines is “no safety concerns” (Tacket et al., 1998a, 2000; Kapusta et al., 1999). The production of vaccine antigens in plants can be achieved through, either stable expression or transient expression systems. The stable genetic transformation produces a genetically engineered plant producing the antigen, and this plant can be propagated either asexually through stem cuttings or sexually through seeds (Tacket et al., 1998b, 2000). On the other hand, transient expression uses recombinant plant virus that carries the vaccine gene and directs the plant to produce the antigen via systematic infection (Koprowski and Yusibov, 2001). Tomato is good alternative for edible vaccines and was used to express orally immunogenic respiratory syncytial virus (RSV) fusion (F) protein in the fruit (Sandhu et al., 2000). Banana is also another good alternative for edible plant vaccines since it is widely grown and transformation has been reported (May et al., 1995). Potato is considered a good model for edible vaccines and the first edible vaccine was tested in potatoes (Tacket et al., 1998b). However, from an economic point of view, it would be better if major crops such as soybean, alfalfa, or corn can also be made efficient plant systems for recombinant antigen protein production (Sanford et al., 1993). Enterotoxigenic bacteria such as *Escherichia* and Cholera cause diarrhea due to the secretion of toxins that specifically bind to G_M1_ gangliosides present on epithelial cell surfaces of small intestine (Sixma et al., 1991). Cholera toxin (CT) and *E. coli* liable toxin (LT) are homologous multi-subunit proteins in which the non-toxic B subunit mediates G_M1_ and thus can be candidates for vaccines that can neutralize toxin activity. Both LT-B (Mason et al., 1998) and CT-B (Arakawa et al., 1998) expressed in transgenic potatoes produced toxin-protective intestinal antibody responses after ingestion, and this shows that plants produced correctly folded proteins and assembled native G_M1_-binding parametric complexes. LT-B potatoes have been used in a clinical study to test the edible plant vaccine (Tacket et al., 1998b). This study successfully shows that transgenic plant material expressing the antigen, are capable of simulating the antibody response in humans. Similarly, several clinical trials have also been performed for other projects, e.g., rabies (Modelska et al., 1998), and *E. coli* O157:H7 (Judge et al., 2004). A step ahead, Wirz et al. (2012) described a fully automated “factory” that uses tobacco plants to produce large quantities of vaccines and other therapeutic biologics within weeks using a biotechnology approach, representing a perfect example and motivation for future endeavors in this direction.

### ANALYSES OF FOOD QUALITY, SAFETY, AND NUTRITIONAL VALUES ARE MORE MEANINGFUL

The field of proteomics has been used to analyze the differences between the nutritional values of food crops through the analysis of their proteomes. Iwahashi and Hosoda (2000) reported that heat stress increased the expression of invertases in tomato fruits, thus increasing their sucrose content and producing sweeter tomatoes. As physiological disorders appears in crop if they are not harvested at right stage and may result in huge economic losses (Lliso et al., 2007; Pedreschi et al., 2007, 2008, 2009), proteomic-based approaches have become useful to detect biomarkers for optimal harvest maturity (Abdi et al., 2002). Analysis of post-harvest withering process in grapes is very critical to produce high quality wines, and thus gel-based proteomics analysis of this process has been employed for improving grape quality (Di Carli et al., 2011). Also understanding the ripening and post-harvest physiology during storage will not only have impact on food quality but also on the optimization of the technological processes involved. Proteomics have investigated the reason that heat treatment for peach fruits will improve the peach fruit quality and shelf-life, and the reason was the differentially expressed proteins that were involved in fruit development and ripening (Zhang et al., 2011a). On the other hand, in cereal industry, proteomics was used for investigating the protein biomarkers for the selection of suitable durum wheat cultivars for pasta making (De Angelis et al., 2008). Flour quality is highly correlated with protein composition and functional quality, thus proteomics can be very useful to identify protein markers for suitable cultivars for flour making (Yahata et al., 2005). The proteomic analysis of wheat kernels for amphiphilic proteins increased the knowledge of the physiological and technological functions of wheat kernels (Amiour et al., 2002). Salt et al. (2005) used 2-DE approach to identify the soluble proteins that play an important role in stabilizing the gas bubbles in dough and influencing the crumbling structure of proteins. Proteomics has also helped in the construction of proteome map investigating the level of protein modification during barley malting and detecting the proteins associated with beer quality (Iimure et al., 2010). Proteomics also had a role in food authenticity, through using sensitive protein biomarkers (Pischetsrieder and Baeuerlein, 2009). Proteomics was used to identify cheaper substitutes for cheaper cultivars of coffee varieties through the use of specific biomarkers (Gil-Agusti et al., 2005). Plant or fruit extracts used in formulas can also be authenticated by the use of protein biomarkers to assess the genuineness of the formula or product (D’Amato et al., 2011; Fasoli et al., 2011).

Food allergens are a great threat to people suffering from such allergies. However, DNA-based techniques have successfully been used but these techniques have limitations as in many instances DNA was completely absent while high quantities of allergy triggering proteins were still present, as in the case of egg white (Popping and Godefroy, 2011). Proteomics is a crucial field for sensitively detecting and quantifying food allergens. A combination of 2-DE and IgE reactive proteins using an allergic patient’s sera has been applied as an approach to characterize the allergenicity of food proteins (Akagawa et al., 2007; Pischetsrieder and Baeuerlein, 2009). Through a proteomics experiment, in which extracted sesame seed proteins were separated by 2-DE followed by immuno-labeling with individual patient sera from 20 patients with sesame seed allergy, four allergen including 7S vicilin-type globulin, 2S albumin seed maturation protein, and embryogenic abundant protein were identified in this study (Beyer et al., 2002). Petersen et al. (2006) compared the allergenic potency of maize pollen and the native grass *Phleum pratense* using 2-DE followed by immuno-blotting, and found that maize pollen showed less allergic response in comparison to the native grass due to lower allergen content and lower allergic groups found in maize pollen. Herndl et al. (2007) also studied apple allergen using 2-DE with IgE immune-blotting and identified four new apple allergens known as Mal d 1, Mal d 2, Mal d 3, and Mal d 4. Proteomic analysis of rice leaf, root, and seed showed the presence of many allergenic proteins in the seeds, which implicate the uses of proteomic analysis of foods for the presence of allergens (Koller et al., 2002). Shotgun proteomics was also used to characterize the allergenicity of certain foods (Chassaigne et al., 2007; Heick et al., 2011b). The generated information is key for targeted approaches, such as selective reaction monitoring (SRM), which not only detect the allergen but also quantify it (Heick et al., 2011a; Lutter et al., 2011). Recently, multi-allergen detection based on an SRM approach was used in the detection of seven allergic foods in bread, five of which are plant origin (Heick et al., 2011a). None-the-less, once a protein of a specific gene or gene families of allergen is confirmed, its expression can be silenced through biotechnological approaches for a safer human consumption of that food (Thelen, 2009).

Recently, proteomics has been used to investigate “plant-based bioactives” to improve the nutritional value of food crops. Bioactives are the peptides that are released either during digestion by the host enzymes or during food processing and ripening by microbial enzymes (Brambilla et al., 2009). Bioactives were reported from different plant sources, such as wheat, rice, maize, soybean, mushrooms, pumpkins, and sorghum (Möller et al., 2008). Soybean bioactive peptides, such as lunasin, Bowman–Birk inhibitor, lectin, and beta-conglycinin, have attracted the attention of researchers who study their antioxidant activities (de Lumen, 2005) to treat oxidative stress in the future (Kussmann et al., 2010). Lupin also contains alpha and beta-conglutins as storage proteins and appears to have bioactive effects (Brambilla et al., 2009).

### BIOFUEL CROP SCIENCE IS ON RIGHT TRACK TO GET BENEFITTED BY PROTEOMICS

Biofuels are obtained primarily from plant biomass, and are believed to have the capacity in the future for substituting fossil fuels for sustainable bioenergy needs (Yuan et al., 2008). Uses of biofuels make a balance between the consumption and release of CO_2_ in the atmosphere. Biofuels, unlike fossil fuels, are made from clean renewable resources such as plants, algae, or photoautotrophic microbes (Schnoor, 2010; Somerville et al., 2010). As the transition from the use of prevalent fossil fuels to the renewable energy resources is a complicated procedure comprising scientific and socio-economic problems (Kullander, 2010), it is very important to shift from using the first generation biofuel crops, such as sugar cane or corn, to the second generation biofuel crops, such as *Miscanthus*, and Cordgrass for the production of bioethanol from lignocellulosic materials found in plants to make a swift change to the most recent and third generation biofuel organisms such as photoautotroph microbes and microalgae. Currently, maize, sugarcane, and rapeseed are among major the crops that are being used for biofuels. One good example of including a new biofuel crop to the list that is under investigation is African grain sorghum. It has been used as food and feed and is now gaining much attention as energy crop (Calviño and Messing, 2012). The *in vitro* suspension culture of sorghum and the characterization of its cell secretome using 2-DE and MS/MS have been studied (Ngara et al., 2008; Ngara and Ndimba, 2011). Another important crop is a non-domesticated oil crop, *Jatropha curcas* L.; has been getting much attention for its oil, which can be converted to bio-diesel, and for its ability to be easily cultivated in arid and semi-arid regions, including wastelands (Johnson et al., 2011). Proteomics has been used to explore the oil body and identify the proteins for oil biogenesis (Liu et al., 2009; Yang et al., 2009). These proteins can be used to employ phylogenetic and molecular breeding strategies in the improvement of this crop (Johnson et al., 2011). *Populus trichocarpa* is a tree model system for energy crops (Singh et al., 2011). Kalluri et al. (2009) used a proteomic, a LC-MS/MS-based approach, and discovered new potential candidate genes in xylem tissue that play an important role in cell wall biosynthesis in addition to cellulose synthase, sucrose synthase, and polygalacturonase. In this way, the use of proteomics to identify candidate proteins (and genes) to improve energy crops for their growing on marginal lands, cheaper breakdown of cellulose and increased total biomass will be reflected in the yield and quantity of their biofuel production capabilities. *Chlamydomonas reinhardtii* is considered as a model system for photoautotrophic growth and lipid and hydrogen production. As these unicellular green algae has been studied and sequenced in many laboratories, now it serves as a model of choice for physiological, ecophysiological, and economical study for the production of biofuels (Huang, 1986; Merchant et al., 2007). The remarkable metabolism of *Chlamydomonas* for energy productions was observed based on its proteomic investigations (Wienkoop et al., 2010). During the investigation, the metabolism showed pronounced effects of carbon concentrating mechanism, which makes the CO_2_ more available for Calvin cycle using carbonic anhydrases. Nearly, 12 isoforms of carbonic anhydrases (CAH4) were found in *Chlamydomonas*, and five isoforms were measured with targeted proteomics and revealed the differences of theses isoforms in respect of concentration patterns (attomole/1000 cells). The mitochondrial isoform of CAH4 showed a very high dynamic range and high activity under the limiting conditions of CO_2_ (Wienkoop et al., 2010)_._ This indicates to the significant role of carbonic anhydrases in CO_2_-sensing pathways in higher plants as well as microalgae, and this novel information improves our understanding and can be used to enhance CO_2_ fixation mechanisms for better biomass production and for increasing the efficiency of biofuel productions irrespective of plants or microalgae (Hu et al., 2009; Moroney et al., 2011; Xue et al., 2011).

### DEVELOPMENT OF PROTEOMICS-BASED FUNGICIDES IS A POSSIBILITY

This possibility relies on the hypothesis that the majority of drug targets are proteins and the proteomics can provide the candidate proteins involved in a specific biological mechanism. Several changes in the design of chemical fungicides are being undertaken by the scientific research community by summarizing the available genomic and proteomic information. Moreover, bioinformatics may come to help in predicting a protein as a fungicide. Biosynthetic fungicide design that is disease-associated target oriented has been established as a new focus in fungicide development (Collado et al., 2007; Acero et al., 2011). However, this field is mostly at its beginning stage but the fungicide design and selection based on target identification information utilizing proteomics experiments is going to change the market in the next 10 years (Garrido et al., 2010; Acero et al., 2011). In depth proteomic and genomic studies of fungal infection biology are a pre-requisite of such projects. The use of modified natural compounds provides a potential species-specific method of controlling plant pathogens by the specific inhibition of those proteins involved in the infection cycle (Pinedo et al., 2008). The use of these compounds minimizes their environmental impact if they are biodegradable, possesses high specificity, and have the further advantage of poor penetration into the food chain. In short, such an application of chemo-genomics to protein targets is named “chemo-proteomics,” although a more explicit definition is target related affinity profiling (TRAP), defined as the use of biology to inform chemistry (Beroza et al., 2002). The accumulation of proteomic information about fungal plant pathogens may be an incentive to the development of new and environmentally friendly fungicides. One of the most promising biotechnologies downstream of proteomics is the use of specific peptide sequences that are able to modify protein activities in the pathogen. One encouraging strategy to combat fungal diseases in the field is the use of a novel chemical proteomics tool called activity-based protein profiling (ABPP; Richau and van der Hoorn, 2010). This technology reveals the activities of proteomes, and that is why understanding the involved biological processes is so crucial. A small-molecule fluorescent probe is used in ABPP; the probe irreversibly reacts with the catalytic sites of catalytic subunits in an activity-dependent manner. By using fluorescent protein gels, the protein activities can be quantified to study these activities *in vitro* and *in vivo *(Gu et al., 2010).

## CONCLUSION

During the recent past, world agriculture has come under more climatic variability along with less arable land availability per person, which compounds the stress situation on producer groups. In the present scenario, pressure is building upon the plant breeders and plant biologists to come up with “smart crop varieties” that are better suited genotypes with the ability to withstand a wider range of climatic variability to tackle the food insecurities of future generations along with maintaining/exceeding quality parameters. Conventional plant breeding approaches, which have played a key role during the green revolution in the 20th century, feel handicapped in the 21st century because modern plant breeders require precise gene modifications with a gene tracking system for the modified trait. In this post-genomic era, the integration of proteomics into the field of crop science will certainly enrich genome annotation efforts and accelerate the development of crop models for the elucidation of gene functions influencing phenotypes for the success of field crops. The only caveat to the application of proteomics in biotechnology programs is that the genetic modification should be expressed at protein level. Progress made with the help of various -omics approaches along with the creation of a wider gene pool by utilizing modern biotechnological tools is the best approach to improve crop productivity for meeting food production goals by 2050.

## Conflict of Interest Statement

The authors declare that the research was conducted in the absence of any commercial or financial relationships that could be construed as a potential conflict of interest.
